# Transapical epicardial temporary pacemaker lead implantation in patients with transapical TAVI

**DOI:** 10.1136/openhrt-2025-003960

**Published:** 2026-08-20

**Authors:** Chunshui Liang, Mingwen Li, Zezhou Feng, Peiqun Zhao, Yingbin Xiao, Ruiyan Ma, Zhao Jian

**Affiliations:** 1 Army Medical University Xinqiao Hospital Department of Cardiovascular Surgery, Chongqing, Chongqing, China

**Keywords:** Heart Valve Prosthesis Implantation, Cardiac Catheterization, Heart Valve Prosthesis

## Abstract

**Objective:**

This study aimed to evaluate the safety and efficacy of transapical epicardial temporary pacemaker lead implantation compared with the conventional transvenous approach in patients undergoing transapical transcatheter aortic valve implantation (TAVI).

**Methods:**

In this single-centre retrospective study, 399 patients who underwent transapical TAVI between December 2018 and August 2024 were included. Of these, 298 received transapical epicardial temporary pacing leads and 101 received transvenous leads. Primary outcomes included pacemaker-related cardiac tamponade, valve displacement and mortality. Secondary outcomes encompassed perioperative complications, permanent pacemaker implantation and postoperative adverse events.

**Results:**

No cases of cardiac tamponade, valve displacement or mortality related to temporary pacing were observed in either group. Poor lead contact occurred in one patient (0.3%) in the transapical group versus three patients (3.0%) in the transvenous group (p=0.05). Postoperative permanent pacemaker implantation was required in 12 transapical patients (4.0%) and three transvenous patients (3.0%, p=0.86).

**Conclusion:**

Transapical epicardial temporary pacing is a safe and effective alternative to transvenous pacing during transapical TAVI, with comparable complication rates and potential technical benefits. Further randomised studies are warranted to validate these findings and explore long-term outcomes.

**WHAT IS ALREADY KNOWN ON THIS TOPIC**

**WHAT THIS STUDY ADDS**

- This large single-centre study demonstrates that transapical epicardial temporary pacing is a safe and effective alternative to transvenous pacing during transapical TAVI, with comparable complication rates and significantly fewer lead contact issues. The technique leverages existing surgical access, provides better lead stability through suture fixation and simplifies pacing lead management.

**HOW THIS STUDY MIGHT AFFECT RESEARCH, PRACTICE OR POLICY**

- These findings support the adoption of transapical epicardial pacing as a routine option in transapical TAVI programmes, potentially reducing pacing-related complications and improving procedural efficiency. The technique may encourage further research into hybrid pacing strategies and influence clinical guidelines for temporary pacing in structural heart interventions.

## Introduction

 Transcatheter aortic valve implantation (TAVI) represents a sophisticated intervention for addressing aortic valve pathologies and has gained extensive application in clinical settings.^1^ The choice of TAVI access route is critical for patients with aortic regurgitation.^2^ While the femoral self-expandable valve approach is undergoing progressive investigation,^3 4^ there is some evidence in the literature supporting the transapical route as a potential option.^5–7^ Rapid ventricular pacing is critical for ensuring the safety and efficacy of TAVI procedures. Currently, this pacing is predominantly achieved through the transvenous implantation of a temporary pacemaker lead into the right ventricle. However, the transapical approach to TAVI, which involves direct exposure of the left ventricular apex, introduces a novel opportunity for epicardial pacing. Transapical epicardial pacing directly influences the left ventricle, theoretically enabling enhanced control over pacemaker wire, minimising complications and reducing the duration of surgical procedures. Currently, there is a lack of comprehensive clinical reports regarding the implementation of transapical epicardial temporary pacemaker lead implantation in patients undergoing transapical TAVI. In this context, our centre has conducted a series of experiments over the past few years involving the implantation of left ventricular apical pacemaker leads in patients undergoing transapical TAVI, during which we have amassed considerable experience and synthesised our findings.

## Methods

### Study population

This study is a retrospective, non-randomised, controlled investigation conducted at a high-volume, single-centre institution. The cohort comprises patients who underwent TAVI at our cardiac surgery centre between December 2018 and August 2024. A total of 399 patients received TAVI (306 cases were transapical approach and 93 cases were femoral approach), with 298 using transapical pacemaker leads (all were transapical approach) and 101 employing transvenous pacemaker leads (eight cases were transapical approach and 93 cases were femoral approach).

### Data variables and definitions

#### Baseline characteristics

Demographic and clinical baseline characteristics were obtained from the department of cardiovascular surgery at Xinqiao Hospital, affiliated with the Army Medical University. Chronic lung disease was defined as either chronic obstructive pulmonary disease or pulmonary fibrosis necessitating medical intervention. A critical preoperative state was characterised by the requirement for intensive care or continuous invasive monitoring of vital parameters prior to undergoing TAVI. Extracardiac arteriopathy was characterised by the presence of significant stenosis in the carotid, subclavian, iliofemoral or femoral arteries. Dialysis was defined as any haemodiafiltration procedure conducted prior to TAVI. The term perioperative antithrombotic drugs encompassed the administration of aspirin, P2Y purinoceptor 12 (P2Y12) platelet inhibitors, warfarin, low molecular weight heparin and non-vitamin K oral anticoagulants within 24 hours before or after the operation. All implanted prosthetic valves were of the self-expandable type. To minimise confounding factors related to the TAVI approach and the specific valve types used, we focused our clinical outcome analysis specifically on pacemaker-related complications, including pericardial tamponade, valve displacement, mortality and pacing lead malfunction.

#### Implanting method of temporary pacemaker leads

##### Transvenous pacemaker implantation

A 5F vascular sheath was inserted via the jugular or femoral vein, and a standard 5F PACEL Bipolar Pacing Catheter (St. Jude Medical, Minnetonka, Minnesota, USA) was advanced through the vascular sheath, navigating through the superior or inferior vena cava, right atrium and tricuspid valve to reach the right ventricle under fluoroscopic guidance. The catheter was subsequently connected to an external temporary pacing generator.

##### Transapical pacemaker implantation

**Figure 1:**
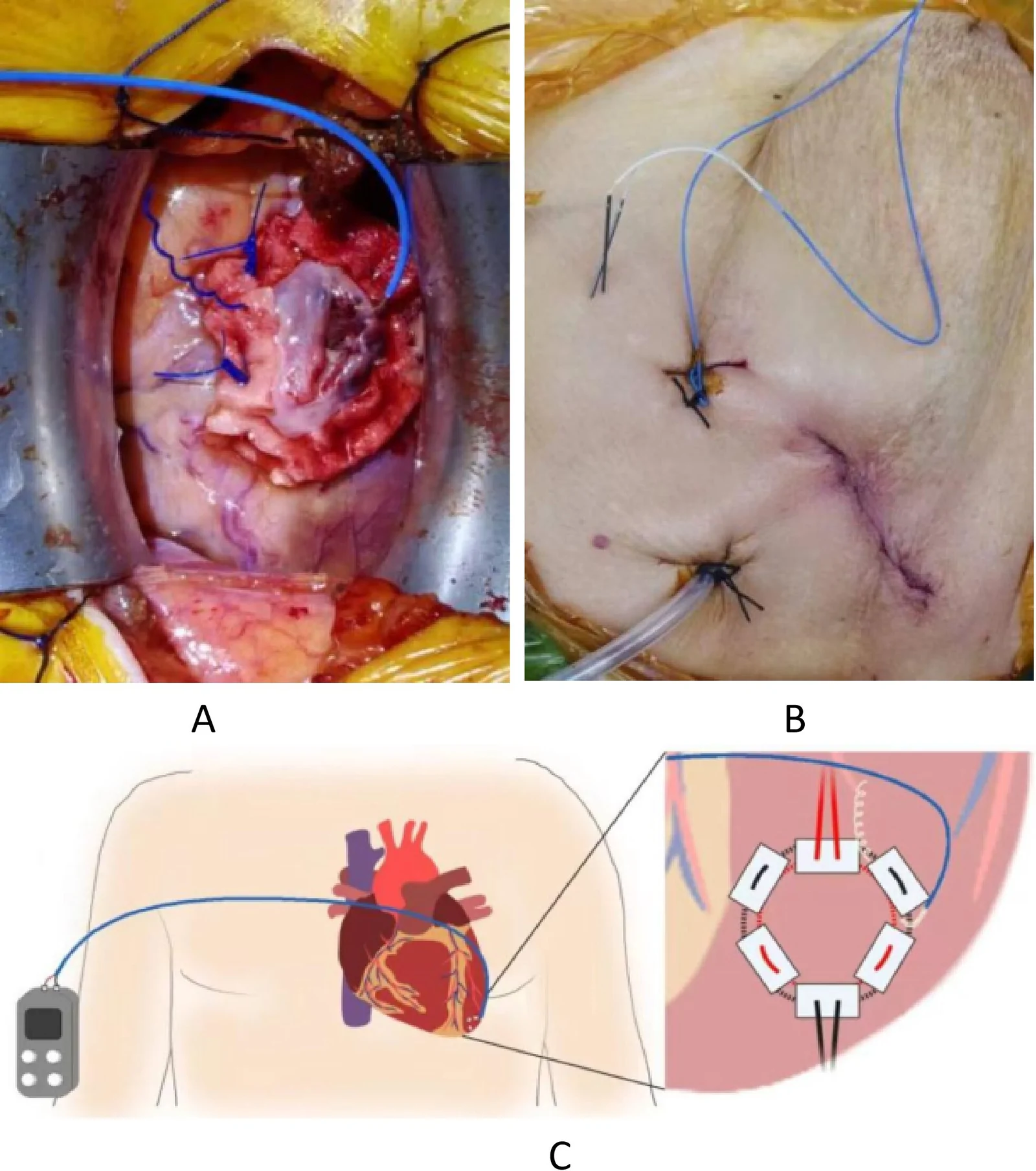
Schematic and intraoperative view of transapical epicardial pacemaker lead placement. (**A**) Intraoperative suturing of the temporary pacing lead. (**B**) Postoperative fixation of the temporary pacing lead. (**C**) Schematic diagram illustrating suture fixation of the temporary pacing lead at the left ventricular apex.

After determining the body surface position of the cardiac apex using preoperative CT, intraoperative fluoroscopy and cardiac ultrasound, the apex of the heart was exposed. A hexagon of appropriate size was created by suturing three adjacent sides to the apex using 3–0 Prolene with a gasket. Subsequently, another 3–0 Prolene with a gasket was sutured approximately 3–4 mm outside the initial gasket to complete the hexagon. The exit points of the two 3–0 Prolene sutures were positioned opposite each other, and a temporary pacemaker lead was sewn between them. Finally, the distal end of the pacemaker lead was secured using 4–0 Prolene (figure 1).

### Outcome measures

The primary outcome measures included temporary pacemaker-related cardiac tamponade, valve displacement and mortality. Cardiac tamponade associated with a temporary pacemaker was defined as bleeding from the suture on the pacemaker lead resulting in haemodynamic compromise within 7 days post implantation. Valve displacement related to the temporary pacemaker was characterised by valve shift during release due to inadequate pacemaker lead contact when rapid ventricular activation is necessitated. A death associated with a temporary pacemaker was classified as a severe complication attributable to the pacemaker lead, resulting in fatality. Additional outcome measures included the incidence of other perioperative tamponade, postoperative permanent pacemaker implantation, suboptimal pacemaker lead contact, postoperative stroke and postoperative myocardial infarction occurring within 30 days following TAVI.

### Statistical analysis

Numerical variables were presented as means and SD, while categorical variables were reported as counts and percentages. The analysis of categorical variables was conducted using the χ^2^ test or Fisher’s exact test, as appropriate. Continuous data were analysed using the independent samples t-test. All statistical analyses were performed using IBM SPSS Statistics V.27.

## Results

### Baseline characteristics

As illustrated in table 1, the cohort receiving transvenous temporary pacemaker leads exhibited a higher proportion of female patients (44.6% vs 32.6%, p=0.03), but a lower prevalence of hypertension (25.7% vs 39.9%, p=0.01) and coronary heart disease (9.9% vs 19.8%, p=0.02). The majority of patients used warfarin for anticoagulation therapy post TAVI. Despite the higher proportion of low molecular weight heparin usage in the transvenous temporary pacemaker lead group (91.1% vs 68.8%, p<0.001), the overall use of any anticoagulant was comparable between the groups (99.3% vs 99.0%, p=0.58).

**Table 1:** Patient and procedural characteristics according to implanting method of temporary pacemaker leads during TAVI

		Transapical temporary pacemaker lead (n=298)	Transvenous temporary pacemaker lead (n=101)	P value
Age, years		70.1±5.3	70.5±4.8	0.54
Female sex		97 (32.6%)	45 (44.6%)	0.03
Body mass index, kg/m^2^		23.4±3.4	23.8±3.8	0.34
Smoke		120 (40.3%)	39 (38.6%)	0.77
Prior stroke		28 (9.4%)	3 (3.0%)	0.06
Gastrointestinal haemorrhage		16 (5.4%)	4 (4.0%)	0.58
Chronic lung disease		79 (26.5%)	17 (16.8%)	0.05
Hypertension		119 (39.9%)	26 (25.7%)	0.01
Atrioventricular block	LBBB	14 (4.7%)	5 (5.0%)	0.32
	RBBB	12 (4.0%)	1 (1.0%)	
	II°	1 (0.3%)	1 (1.0%)	
	III°	5 (1.7%)	0 (0%)	
Permanent pacemaker		2 (0.7%)	0 (0%)	0.56
Atrial fibrillation		33 (11.1%)	9 (8.9%)	0.54
Coronary heart disease		59 (19.8%)	10 (9.9%)	0.02
Previous PCI		11 (3.7%)	5 (5.0%)	0.58
Recent myocardial infarction		0 (0%)	0 (0%)	/
Diabetes mellitus		36 (12.1%)	13 (12.9%)	0.83
Chronic renal failure		35 (11.7%)	10 (9.9%)	0.61
Dialysis		3 (1.0%)	0 (0%)	0.42
Extracardiac arteriopathy		6 (2.0%)	1 (1.0%)	0.81
Tumour history		7 (2.3%)	0 (0%)	0.27
Critical preoperative state		6 (2.0%)	2 (2.0%)	0.67
Hyperlipidaemia		21 (7.0%)	13 (12.9%)	0.07
NYHA class	II	26 (8.7%)	6 (5.9%)	0.67
	III	221 (74.2%)	77 (76.2%)	
	IV	51 (17.1%)	18 (17.8%)	
Previous cardiac surgery	Aortic valve	5 (1.7%)	2 (2.0%)	0.31
	Other	0 (0%)	1 (1.0%)	
Perioperative antithrombotic drugs	Aspirin	12 (4.0%)	2 (2.0%)	0.51
	P2Y12 inhibitors	4 (1.3%)	1 (1.0%)	0.63
	Warfarin	295 (99.0%)	99 (98.0%)	0.60
	LMWH	205 (68.8%)	92 (91.1%)	<0.001
	NOACs	0 (0%)	0 (0%)	/
	Warfarin/LMWH/NOACs	296 (99.3%)	100 (99.0%)	0.58

Values are n (%) or mean ± SD, depending on variable distribution.

LBBB, left bundle branch block; LMWH, low molecular weight heparin; NOACs, non-vitamin K oral anticoagulants; NYHA, New York Heart Association functional class; PCI, percutaneous coronary intervention; P2Y12, P2Y purinoceptor 12; RBBB, right bundle branch block; TAVI, transcatheter aortic valve implantation.

### Outcomes

Cardiac tamponade, valve displacement and mortality associated with the use of a temporary pacemaker, which constituted the primary outcome, were not observed in either group. Additionally, there were no occurrences of perioperative tamponade or perioperative myocardial infarction. During the valve implantation procedure, one patient in the transapical group experienced poor contact, which was promptly resolved. Conversely, in the transvenous group, three patients encountered poor contact, necessitating a longer adjustment period, but no statistical significance (p=0.05). Furthermore, two patients in the transapical group developed cerebral infarctions. In the transapical group, 12 patients received permanent pacemaker implants postoperatively, whereas in the transvenous group, three patients underwent the same procedure (refer to table 2).

**Table 2:** Periprocedural and postprocedural clinical outcomes according to implanting method of temporary pacemaker leads during TAVI

		Transapical temporary pacemaker lead (n=298)	Transvenous temporary pacemaker lead (n=101)	P value
Temporary pacemaker-related	Cardiac tamponade	0 (0%)	0 (0%)	/
	Valve displacement	0 (0%)	0 (0%)	/
	Death	0 (0%)	0 (0%)	/
Poor pacemaker lead contact		1 (0.3%)	3 (3.0%)	0.05
Other perioperative tamponade		0 (0%)	0 (0%)	/
Postoperative permanent pacemaker implantation		12 (4.0%)	3 (3.0%)	0.86
Postoperative stroke		2 (0.7%)	0 (0%)	0.56
Perioperative myocardial infarction		0 (0%)	0 (0%)	/

Cardiac tamponade, vascular complication and perioperative myocardial infarction are within 7 days of the procedure. Postoperative stroke periprocedural death and permanent pacemaker implantation are within 30 days of the procedure. Both cases of death related to temporary pacemaker occurred due to cardiac tamponade.

TAVI, transcatheter aortic valve implantation.

## Discussion

The traditional transjugular temporary pacemaker lead implantation remains the predominant technique employed in TAVI procedures.^8^ This method is pivotal during the valve deployment phase; however, improper management can result in significant complications, including valve displacement, myocardial perforation and pericardial tamponade.^9–11^ Furthermore, patients with a heightened risk of conduction block—such as those with right bundle branch block, left anterior hemiblock, second-degree atrioventricular block or extensive calcification in the conduction system—are predisposed to developing third-degree atrioventricular block postoperatively, necessitating the implantation of a permanent pacemaker.^12 13^ Maintaining optimal contact with the temporary pacemaker lead is crucial to ensure stable heart rates and prevent serious adverse events.

Numerous studies have explored alternative methods to address the limitations associated with the traditional transjugular pathway.^8 14–18^ For instance, simple left ventricular temporary pacing has been employed to mitigate the risk of pericardial tamponade, a complication observed with the transvenous route.^8^ Furthermore, the utilisation of a permanent pacemaker lead, as opposed to a temporary one, not only diminishes the risk of pericardial tamponade but also enhances the stability of the temporary pacemaker lead, thereby reducing the incidence of adverse outcomes.^14^ Alternatively, partial inflation of the balloon of the pacing lead can be used to reduce right heart perforation without reducing pacemaker lead contact. Additionally, various other techniques are used to achieve similar outcomes.^15^ For instance, intracoronary temporary rapid ventricular pacing has proven effective during valve-in-valve TAVI with coronary protection via the ‘chimney technique,’ particularly following unsuccessful attempts at retrograde left endocardial temporary rapid ventricular pacing.^16^ Real-time three-dimensional transesophageal echocardiography guidance has the potential to become the preferred modality for the perioperative implantation of transvenous temporary pacemakers in patients undergoing cardiac surgery, as opposed to traditional fluoroscopy.^17^ Additionally, it has been proposed that using an upper extremity approach may offer superior safety and efficacy compared with the transfemoral approach in TAVI procedures.^18^

In all cardiac surgeries involving thoracotomy, we routinely place temporary pacemaker leads on the epicardium for ventricular pacing. These leads are secured with sutures to prevent displacement and ensure functionality, while also providing haemostasis. During transapical TAVI, the epicardium is exposed, facilitating the skilled and safe insertion of temporary pacemaker leads. Our study indicated no instances of serious cardiac bleeding, pericardial tamponade or valve displacement due to poor contact when the temporary pacemaker leads were implanted transapically. Our study confirmed that the transapical temporary pacemaker lead was not inferior to the transvenous approach and presented several advantages: (1) it allows for a straightforward routine procedure for surgeons, (2) it reduces invasiveness by avoiding vein puncture, (3) it is time-efficient, as cardiac sewing is faster than the transvenous method, (4) it offers better stability through suture fixation, (5) it is easily adjustable in cases of poor contact, (6) it lowers the risk of bleeding, with the ability to visually confirm and stop bleeding post-surgery and (7) the lead can be retained for long-term use postoperatively, (8) the technique does not impact the patient’s ability to mobilise out of bed.

However, it is applicable exclusively to patients undergoing transapical TAVI. It is important to acknowledge certain limitations within our study. Notably, the surgical approaches between the two groups differ, with the transvenous group including both transapical and transfemoral TAVI procedures. Consequently, the potential influence of varying surgical approaches cannot be excluded. To substantiate our findings, further research incorporating multicentre studies, larger sample sizes and randomised controlled trials is necessary.

### Study limitations

The primary limitation of this study lies in the inclusion of patients undergoing different TAVI access routes (transapical and transfemoral) within the transvenous pacing group. Although we focused on pacemaker-related complications to minimise confounding effects, the potential baseline risk differences introduced by the distinct surgical approaches themselves cannot be fully excluded, which may have affected the direct comparability between the groups. Furthermore, the observational and retrospective nature of this study carries an inherent risk of selection bias. Additional constraints include its single-centre design and a relatively limited sample size, which may restrict the generalisability of the findings. Finally, non-clinical factors, such as economic considerations and the preferences of both physicians and patients, might have introduced further bias in the patient selection process.

## Conclusions

Transapical epicardial temporary pacing is a safe and effective alternative to transvenous pacing during transapical TAVI, with comparable complication rates and potential technical benefits. Further randomised studies are warranted to validate these findings and explore long-term outcomes.

## Data Availability

Data are available upon reasonable request.
